# Magnetic resonance imaging for distinguishing ovarian clear cell carcinoma from high-grade serous carcinoma

**DOI:** 10.1186/s13048-016-0251-x

**Published:** 2016-07-04

**Authors:** Feng-Hua Ma, Jin-Wei Qiang, Guo-Fu Zhang, Hai-Ming Li, Song-Qi Cai, Ya-Min Rao

**Affiliations:** Department of Radiology, Obstetrics & Gynecology Hospital, Shanghai Medical College, Fudan University, 419 Fangxie Road, Shanghai, 200011 Huangpu District China; Department of Radiology, Jinshan Hospital, Shanghai Medical College, Fudan University, 1508 Longhang Road, Shanghai, 201508 Jinshan District China; Department of Pathology, Obstetrics & Gynecology Hospital, Shanghai Medical College, Fudan University, Shanghai, 200011 China

**Keywords:** Ovary, Clear cell carcinoma, High-grade serous carcinoma, Magnetic resonance imaging

## Abstract

**Background:**

To compare the magnetic resonance imaging (MRI) features of ovarian clear cell carcinoma (CCC) and high-grade serous carcinoma (HGSC), to distinguish CCC from HGSC.

**Methods:**

MRI features (laterality, shape, size, configuration, papillary projection, signal intensity, enhancement, peritoneal implant, lymphadenopathy, ascites) of 40 tumors in 37 patients with CCC, confirmed by surgery and pathology, were compared with those of 62 tumors in 40 patients with HGSC. Statistical analysis was performed using Mann-Whitney and Fisher’s exact tests.

**Results:**

There was a statistically significant difference in the mean maximum diameter, laterality, and FIGO stage (*P* = 0.002, *P* < 0.001, *P* < 0.001, respectively) between CCC and HGSC. Compared to HGSCs, CCCs were more frequently oval (30/40, 75 % vs 12/62, 19 %; *P* < 0.001), more often cystic (21/40, 53 % vs 8/62, 13 %; *P* < 0.001) and unilocular (23/29, 79 % vs 7/31, 23 %; *P* < 0.001), had T1-hyperintense cystic components more often (18/29, 62 % vs 5/29, 17 %; *P* < 0.001), had larger papillary projections (5.13 ± 0.4 cm vs 2.91 ± 0.3 cm; *P* < 0.001), were peritoneally implanted less frequently (*P* = 0.001) and had fewer ascites (*P* < 0.001).

**Conclusions:**

CCC typically showed an oval, unilocular cystic mass with large papillary projection and T1-hyperintense cystic components. MRI could be helpful for distinguishing CCC from HGSC.

## Background

Clear cell carcinoma (CCC) has recently emerged as the second most common type of epithelial ovarian cancers (EOCs), representing 5–25 % of ovarian carcinomas. CCC is a highly distinct entity from ovarian high-grade serous carcinoma (HGSC), which is the most frequent subtype of EOCs [1, 2]. Compared with ovarian HGSCs, CCCs present at a younger age, have a higher incidence of stage I disease, rarely occur bilaterally, and often consist of large pelvic mass in association with endometriosis [3–5]. It has been widely assumed that most CCCs appear resistant to conventional chemotherapeutic agents and have relatively poorer prognoses than other EOC subtypes when they are in advanced stages [4–6]. However, patients with early stage CCC might have better prognoses than patients with HGSC and might not require any adjuvant therapy [4, 7, 8]. Given their distinctive biological and clinical features, the therapeutic strategy for ovarian CCC is different from that for HGSC in the 2014 version of the National Comprehensive Cancer Network (NCCN) guidelines [9]. Therefore, early detection and accurate preoperative diagnosis of ovarian CCC are of critical importance for an optimal therapeutic strategy in this epoch of precision medicine.

Owing to multiplanar imaging and its superior capability for soft tissue contrast, magnetic resonance imaging (MRI) has the ability to characterize complex adnexal masses and could contribute to the correct identification of CCC [10, 11]. To date, only a few case reports and a small sample of MRI studies concerning CCC have been available [12, 13]. Furthermore, differentiation between CCC and HGSC has not yet been investigated. Therefore, the purposes of this study were to investigate the characteristic features of CCC and to evaluate MRI for distinguishing CCC from HGSC.

## Methods

### Clinical data

This retrospective study was approved by the two institutional review boards of Jinshan Hospital and Obstetrics & Gynecology Hospital, Shanghai Medical College, Fudan University, China. The informed consent requirement was waived. From February 2008 to December 2014, patients suspected of ovarian tumors by gynecologic examination, biomarkers, US or CT were enrolled in a MRI study project of ovarian tumor. Among 530 cases proven by surgery and histology, a total of 37 patients with 40 pure CCCs, confirmed by surgery and histopathology, were found. We excluded 6 patients with CCC mixed with other types of EOC and and patients with recurrent tumors were also excluded. Pelvic masses were found in 27 (73 %) patients during routine physical examinations, and the remaining 10 patients presented with non-specific symptoms, such as abdominal pain (22 %) and loss of weight (5 %).

For comparison, a total of 40 patients with 62 primary HGSCs were randomly selected from the same databases during the same period as the control group. All of the patients in both groups underwent surgery (laparoscopy in 4 cases, laparotomy in 73 cases) within two weeks after completing MRI scans. The tumors were staged according to the International Federation of Gynecology and Obstetrics (FIGO) 2013 staging system.

### MRI scanning

Images were acquired with 1.5 T MR scanner (Symphony or Avanto, Siemens, Erlangen, Germany), with a phased-array pelvic coil. The patient lay in the supine position and breathed freely during image acquisition. The following sequences were obtained: axial spin-echo (SE) T1-weighted imaging (T1WI) (repetition time [TR]/echo time [TE] = 761/10 ms); T1WI flash 2D with fat saturation [TR/TE = 196/2.9 ms]; turbo SE T2-weighted imaging [T2WI] with and without fat saturation [TR/TE = 8000/83 ms and 4000/98 ms, respectively], and turbo SE sagittal and coronal T2WI (TR/TE = 8000/98 ms). Contrast-enhanced flash 2D T1WI with fat saturation (TR/TE = 196/2.9 ms) was performed on the axial and sagittal planes after the intravenous administration of 0.2 mmol/kg Gadopentetate dimeglumine (Gd-DTPA, Magnevist; Bayer Schering, Berlin, Germany) at a rate of 2–3 ml/second. The scanning parameters were as follows: slice thickness 5 mm; gap 1.5 mm; matrix 256 × 256; field of view 20–25 cm × 34 cm; and excitations 4. The scanning range was from the inferior public symphysis to the renal hilum and extended beyond the dome of the tumor in cases with large masses.

### Image analysis

The MRIs were reviewed independently by two radiologists (F.H.M. and J.W.Q.) who had 12 and 31 years of experience, respectively, in gynecological imaging. Both of the radiologists were blinded to the pathological results. Any discrepancies were resolved by consensus. Tumor size, presence and relative signal intensity of a solid and/or cystic component, presence and number of septa, degrees of enhancement and associated findings (ascites, endometriosis, peritoneal implants, lymphadenopathy and distant metastasis) were recorded on both T2WI and contrast-enhanced T1WI. The cystic and solid component was defined as tissue with on enhancement and enhancement after injection respectively. The relative signal intensity and degrees of enhancement of a solid and/or cystic component were referring to the signal intensity and enhancement of outer myometrium. Tumors were classified as predominantly cystic, mixed cystic-solid, or solid. The size of tumor, septa and solid component were measured on the contrast-enhanced T1WI.

### Histopathology

The histopathological evaluation was performed by two pathologists who specialized in gynecological pathology, and the diagnosis was determined by consensus. Data were recorded on a standardized form specifically designed for ovarian tumors and included laterality, tumor size, shape and color, and cystic and solid components.

### Statistical analysis

Statistical analysis was performed with SPSS software, version 17.0 for Windows (SPSS Inc., Chicago, IL, USA). The differences between CCC and HGSC in laterality, shape, configuration, unilocularity, papillary projections, signal intensity on T1WI, enhancement and associated findings were compared using Pearson’s Chi-square test or Fisher’s exact test, and age and size were compared using the Mann-Whitney test. Binary logistic regression was used to assess the predictive value of MRI findings for CCC. A *p*-value less than 0.05 was considered to be statistically significant. The diagnostic performance of the significant MRI features for characterizing ovarian CCC was also determined.

## Results

Ten of 40 CCCs were found to arise from endometriosis, and 4 evolved from borderline clear cell tumors. The clinical features of CCC compared with HGSC are shown in Table 1. There was a statistically significant difference in the maximum diameter, laterality, and FIGO stage (*P* = 0.002, *P* <0.001, *P* <0.001, respectively) between CCC and HGSC. No statistically significant differences were found in age, menopause status, or surgical approach (*P* = 0.231, 0.459, 0.268, respectively) between the two groups.

**Table 1:** Comparison of clinical features between ovarian CCC and HGSC

Clinical features	CCC (*n* = 37)	HGSC (*n* = 40)	*P* value
Mean age	51 ± 1.4	54 ± 1.4	0.231
Mean diameter	11.4 ± 0.7	8.6 ± 0.6	0.002
Laterality			<0.001
Unilateral	34 (91 %)	18 (45 %)	
Bilateral	3 (9 %)	22 (55 %)	
Menopausal status			0.459
Postmenopausal	21 (57 %)	26 (65 %)	
Premenopausal	16 (43 %)	14 (35 %)	
Surgical approach			0.268
Laparotomy	34 (92 %)	39 (98 %)	
Laparoscopy	3 (8 %)	1 (2 %)	
FIGO stage			<0.001
I	23 (62 %)	4 (10 %)	
II	5 (14 %)	6 (15 %)	
III	8 (23 %)	27 (68 %)	
IV	1 (3 %)	3 (7 %)	

*CCC* clear cell carcinoma, *HGSC* high-grade serous carcinoma, *FIGO* International Federation of Gynecology and Obstetrics

**Fig. 1:**
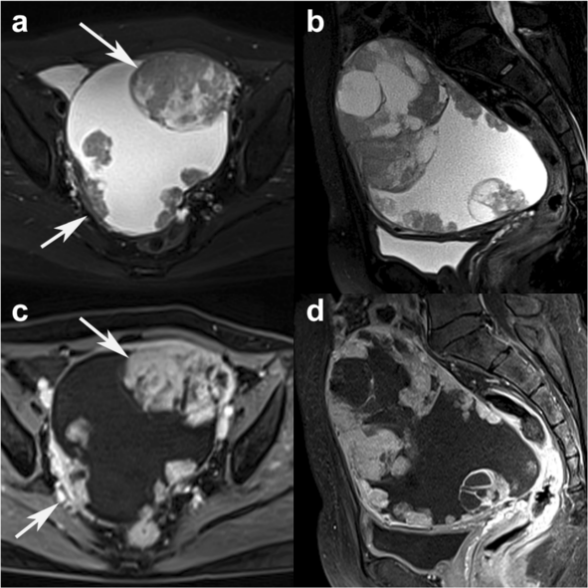
A 53-year-old woman with left ovarian clear cell carcinoma (CCC). Axial and sagittal turbo spin echo (TSE) T2-weighted imaging (T2WI) with fat saturation (FS) (**a**-**b**) show an oval unilocular cystic mass with papillary projections (arrows). Axial and sagittal contrast-enhanced flash 2D T1WI with FS (**c**-**d**) show prominent enhancements in solid components

**Fig. 2:**
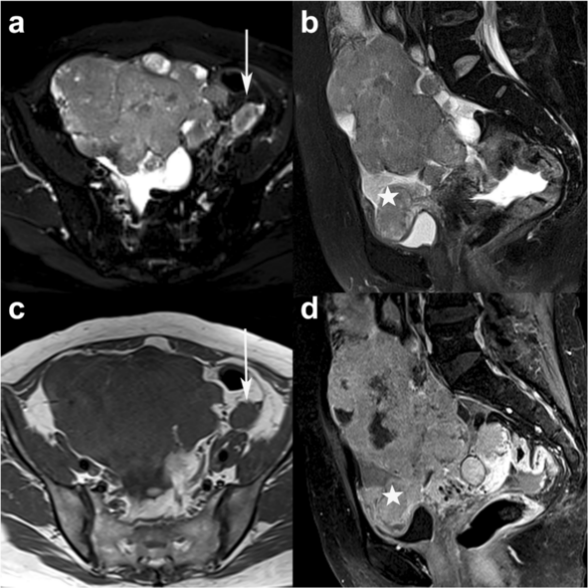
A 50-year-old woman with bilateral high-grade serous carcinoma (HGSC). Axial and sagittal TSE T2WI with FS (**a**-**b**), axial T1WI (**c**) and sagittal contrast-enhanced flash 2D T1WI with FS (**d**) show the irregular solid mass appearing with iso-intensity on T1WI and prominent enhancement on contrast-enhanced T1WI. There are peritoneal implantations (white stars) in the vesico- and rectouterine pouches and lymphadenopathy (arrows) in front of the left iliac vessels (FIGO stage IIIc)

**Fig. 3:**
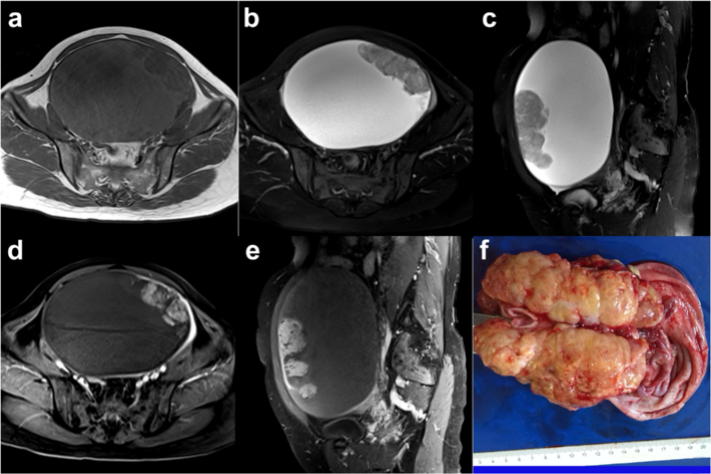
An 81-year-old woman with CCC in the left ovary. Axial SE T1WI, axial and sagittal TSE T2WI with FS (**a**, **b**, **c**) demonstrate a unilocular cystic mass with a large papillary projection, with prominent enhancement on contrast-enhanced flash 2D T1WI with FS (**d**, **e**). Pathologic specimen (**f**) of the mass shows a large papillary projection protruding into the lumen

**Fig. 4:**
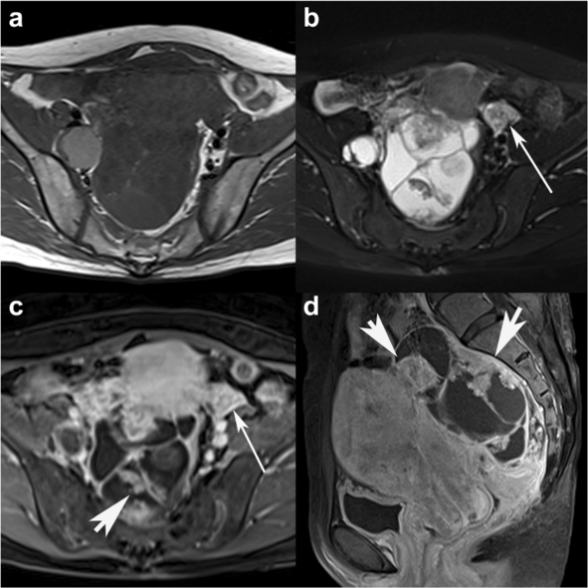
A 42-year-old woman with HGSC in the right ovary. Axial SE T1WI and TSE T2WI with FS (**a**-**b**) show a mulitlocular cystic mass with multiple small papillary projections. Axial and sagittal contrast-enhanced flash 2D T1WI with FS (**c**-**d**) show the prominently enhanced papillary projections (arrowheads) and lymphadenopathy (arrows) beside the bilateral iliac vessels

**Fig. 5:**
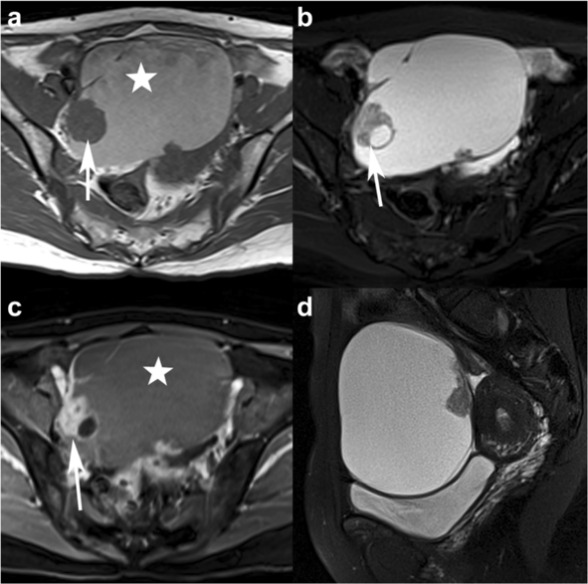
A 49-year-old woman with CCC in the right ovary. Axial SE T1WI, axial and sagittal TSE T2WI with FS (**a**-**c**) demonstrate a unilocular cystic mass with multiple papillary projections (arrows). The signal intensity of the cystic component is high on both T1WI and T2WI (white stars). The papillae show prominent enhancement on contrast-enhanced flash 2D T1WI with FS (**d**)

The MRI features of CCC compared with HGSC are shown in Table 2. Compared to HGSC, CCC appeared to be more frequently oval (30/40, 75 % vs 12/62, 19 %) and less frequently irregular (10/40, 25 % vs 50/62, 81 %), more often cystic (21/40, 53 % vs 8/62, 13 %), less often mixed cystic-solid (8/40, 20 % vs 21/62, 34 % and solid (11/40, 27 % vs 33/62, 53 %), and more often unilocular (23/29, 79 % vs 7/31, 23 %), with significant differences (all *P* < 0.001) (Figs. 1 and 2). Papillary projections were found in 65 % (26/40) of CCCs versus 50 % (31/62) of HGSCs (*P* = 0.073) and were larger in CCCs (5.13 ± 0.4 cm) than in HGSCs (2.91 ± 0.3 cm) (*P* <0.001) (Figs. 3 and 4). The signal of the cystic component on T1WI was hyperintense in 62 % (18/29) of CCCs versus iso- or hypointense in 83 % (24/29) of HGSCs (*P* <0.001) (Fig. 5). The enhancement was mild in 5 %, moderate in 17 % and prominent in 78 % of CCCs versus 3 %, 13 %, 84 % of HGSCs, respectively (*P* = 0.717). There was statistically significant difference between the two groups in peritoneal implantation (*P* = 0.001) and ascites (*P* <0.001). Using binary logistic regression analysis, the most significant predictive features of CCC were a unilocular cystic mass (Odds ratio[OR] = 19.9, 95 % confidence interval [CI]: 5.4–74.1), oval shape (OR = 12.5, 95 %; 4.8–32.4), large papillary projections (OR = 9.5, 95 % CI: 1.2–88.4), and hyperintensity on T1WI (OR = 8.5, 95 % CI: 2.5–28.7).

**Table 2:** Comparison of MRI features between CCC and HGSC

MRI features	CCC (*n* = 40)	HGSC (*n* = 62)	*P* value	Odds Ratio
Shape			<0.001	12.5 (4.8 ~ 32.4)
Oval	30 (75 %)	12 (19 %)		
Irregular	10 (25 %)	50 (81 %)		
Configuration			<0.001	0.2 (0.1 ~ 0.4)
Cystic	21 (53 %)	8 (13 %)		
Cystic-solid	8 (20 %)	21 (34 %)		
Solid	11(27 %)	33 (53 %)		
Unilocular	23/29 (79 %)	7/31 (23 %)	<0.001	19.9 (5.4 ~ 74.1)
Papillary projections	26 (70 %)	31 (50 %)	0.073	——
Size of papillary projection^a^	5.13 ± 0.4	2.91 ± 0.3	<0.001	9.5(1.2 ~ 88.4)
T1-hyperintense cystic component	18/29 (62 %)	5/29 (17 %)	<0.001	8.5 (2.5 ~ 28.7)
Enhancement			0.717	——
Mild	2 (5 %)	2 (3 %)		
Moderate	7 (17 %)	8 (13 %)		
Prominent	31 (78 %)	52 (84 %)		
Peritoneal implantation^b^	1 (3 %)	12 (30 %)	0.001	0.1 (0.01 ~ 0.7)
Lymphadenopathy^b^	7 (19 %)	8 (20 %)	0.905	——
Ascites^b^	13 (35 %)	32 (85 %)	<0.001	0.2 (0.5 ~ 0.4)

*MRI* magnetic resonance imaging; ^a^mean maximal size of papillary projection; ^b^No. (percentage) in 37 patients with CCC and 40 patients with HGSC

Diagnostic performances for the characterization of CCC are listed in Table 3. The combination of any two of four features -- a unilocular cystic mass, oval shape, large papillary projections (≥4 cm) and hyperintensity on T1WI -- yielded sensitivity, specificity, accuracy, positive and negative predictive values, and a positive likelihood ratio for identifying CCC of 90 % (36/40), 87 % (54/62), 88 % (90/102), 82 % (36/44), 93 % (54/58), and 6.92, respectively.

**Table 3:** Diagnostic performance of MRI features for characterizing ovarian CCC

MRI features	Sensitivity (%)	Specificity (%)	Accuracy (%)	PPV (%)	NPV (%)	PLR
Unilocular cystic mass	90 (26/29)	89 (24/31)	83 (50/60)	79 (26/33)	89 (24/27)	8.18
Oval shape	75 (30/40)	82 (51/62)	79 (81/102)	73 (30/41)	84 (51/61)	4.16
Papillary projections (≥3 cm)	85 (24/28)	71 (21/31)	76 (46/59)	71 (24/34)	76 (21/25)	2.93
T1-hyperintense cystic component	62 (18/29)	84 (26/31)	73 (44/60)	78 (18/23)	70 (26/37)	3.88

Data in parentheses are the numbers of masses; *PPV* positive predictive value, *NPV* negative predictive value, *PLR* positive likelihood ratio

## Discussion

Ovarian carcinomas comprise a heterogeneous group of tumors, the four most common subtypes being serous, endometrioid, clear cell and mucinous. In recent years, considerable advances have been achieved in the understanding and identification of the underlying pathogenesis in different subtypes [1, 14]. Our previous study showed that conventional MRI combining DWI may be helpful for differentiating ovarian endometrioid carcinomas from HGSC [15]. Previous studies have indicated that there are different risk factors, origins, genetic alterations, biological behaviors, clinicopathological characteristics and chemotherapy sensitivities between ovarian CCC and HGSC [2–6, 16]. In our clinical practice, we also have found the MRI features between CCC and HGSC may be different. So we try to investigate the characteristic features of CCC and to evaluate MRI for distinguishing CCC from HGSC.

Clinically, patients with CCC are more likely to present with a unilateral (89–95 %), large pelvic mass (12 cm–13.5 cm) and stage I disease (56–63 %) in association with endometriosis (31–48 %) [5, 17, 18]. In contrast, patients with HGSC are more likely to be present with a bilateral (50 %), medium-sized mass (8.6 cm) and advanced stage disease (81 %) [5, 17]. In this study, significant differences were found in unilaterality (91 % vs 55 %), mass size (11.4 cm vs 8.6 cm) and stage I disease (62 % vs 10 %) between ovarian CCCs and HGSCs. Ovarian CCCs were confirmed to be derived from the endometriosis in 25 % of the patients, which was an incidence lower than those in previous studies [17, 18]. A possible explanation for this result was insufficient sampling due to the study not being pathogenesis-oriented. In contrast, most ovarian HGSCs are believed to derive from the tubal intraepithelial lining [19], and only 7 % of cases have histories of ovarian endometriosis [20].

Although a specific histologic type could not be diagnosed on the basis of MR imaging, some imaging features were more common and more suggestive of one or some histologic types. For example, unilocular cystic masses with one or more nodules protruding into the lumen constitute a typical appearance of CCC [12, 13]. This typical appearance was seen in 65 % (26/40) of our CCCs, while multilocular mixed cystic-solid masses with small papillary projections and solid masses were the typical features of HGSCs.

The identification of papillary projections on MR images is important because they are the best predictors of an epithelial tumor and can be correlated with the aggressiveness of the tumor [21]. Histologically, they represent folds of epithelial proliferation growing over a stromal core. Papillary projections in differentiating benign tumors from borderline or malignant counterparts have been reported [21, 22]. However, to our knowledge, no attempts were made to use these projections to distinguish the different subtypes of EOC. In this study, there was no significant difference in the presence of papillary projections between CCC and HGSC. However, the mean size of the papillary projections was significantly larger in CCC than in HGSC (5.13 ± 0.4 cm vs 2.91 ± 0.3 cm), which was inconsistent with the findings of Buy et al [23]. On microscopy, the papillae of HGSC are small, irregular, and hierarchically branching, in contrast with the large, round, and more simplified papillae of CCC [1, 24].

The signal of the cystic components on T1WI was hyperintense in 62 % of CCCs versus iso- or hypointense in 83 % of HGSCs, similar to pelvic muscle or equal to urine. The high intensity of the cystic components, indicating high attenuation on CT, as reported by Choi et al. [13], might be caused by the presence of intracystic hemorrhage from associated endometriosis. We believe that this high intensity could be one of the points for distinguishing CCC from HGSC.

Cystic-solid and solid masses were less common in CCCs (47 %) than in HGSCs (87 %). The enhancement of solid components on contrast-enhancement MRI did not help in distinguishing between CCC and HGSC; however, the shape of mass could be a differentiating feature, with more oval and fewer irregular masses in CCCs than in HGSCs. Associated findings, such as ascites, endometriosis, and peritoneal implantation, might also be important points of distinction between CCC and HGSC. Massive ascites and peritoneal implants were strongly indicative of HGSC, while endometriosis was indicative of CCC.

There were some limitations of our study. First, only a limited number of patients were evaluated, and selection bias was inevitably present due to the retrospective nature of the study. Second, the value of functional imaging, such as diffusion- and perfusion-weighted imaging and spectroscopy, was not investigated. Third, inter-reader variability was not assessed.

## Conclusion

Our preliminary study showed that a large unilocular, cystic mass and papillary projections were the typical features of CCC. The size, laterality, FIGO stage, shape, configuration, unilocularity, T1-hyperintense cystic component, papillary projection size, peritoneal implant and ascites were the ten features that were helpful for distinguishing CCC from HGSC.

## Abbreviations

CCC, Clear cell carcinoma; CI, Confidence interval; EOC, Epithelial ovarian cancers; FIGO, International Federation of Gynecology and Obstetrics; HGSC, High-grade serous carcinoma; NCCN, National Comprehensive Cancer Network; NPV, negative predictive value; OR, Odds Ratio; PLR, positive likelihood ratio; PPV, positive predictive value
